# Planetary Health and Mental Health Nexus: Least Understood and Embraced in Policy Decisions

**DOI:** 10.5334/aogh.4455

**Published:** 2024-07-16

**Authors:** Manasi Kumar, Pim Cuijpers, Pushpam Kumar

**Affiliations:** 1 Institute for Excellence in Global Health Equity, New York University Grossman School of Medicine, New York, USA; 2 Vrije Universiteit Amsterdam, Amsterdam, The Netherlands; 3 United Nations Environment Programme, Washington DC, USA

**Keywords:** Mental health, climate change, biodiversity, behavioral sciences, natural resources, depression, suicidality

## Abstract

Planetary health influences mental health and a better management of climate, biodiversity and pollution has co-benefits of improving mental health outcomes. The recognition and treatment of mental health, however, has been marginalized within environmental and climate change sciences and a greater understanding of the complex underlying processes and societal costs is required to appropriately manage and motivate policy responses.

The paper provides seven recommendations underscoring that public policy developers and implementors need to be aware of the combined costs of inaction – that might accrue from neglecting mental health and environmental sciences– two areas that have been historically marginalized. Improved methodologies in conducting studies on the nature and mental health nexus are needed. The trajectories and models of adaptation and mitigation of climate change and environmental damage can be strengthened through adoption of mental and behavioral sciences approach.

## Introduction

Planetary crisis manifested through rise in temperature, shrinking of green space (or declining biodiversity) and growing pollution and chemicals including plastics inflict heavy health costs. Climate change along with degradation of air, water and land-based resources has serious implications for human health, including mental health impacts which are well documented [1–4].

While working on the nexus of planetary health and human health, as a team of interdisciplinary researchers, we found that while mental health is an important dimension of public health and is a key determinant of human health, it is the least understood and most largely ignored area in public policy including formulation of environmental management. In this piece, first we attempt to highlight the linkages of planetary crisis and mental health; next, we provide a rationale for why it needs attention and finally suggest pathways to internalise these co-benefits of environmental management to attract investment in climate biodiversity, mental health and joint programming involving the fields of mental health and behavioral sciences.

## Climate Change

Climate, loss of green space and rise in pollution particularly are exacerbating public health burden and have an alarming impact on mental health which is not understood by the conventional decision makers at the national level. The Intergovernmental Panel on Climate Change (IPCC) suggests an increase of global surface temperature that is 1.09°C higher in 2011–2020 from 1850–1900, before the industrial revolution [5]. Moreover, average surface temperatures have increased more rapidly since 1970 than in any other 50-year period over at least the last 2000 years [5]. Since weather systems are interconnected, alterations in temperature have cascading effects on various weather phenomena, such as drought, wildfire and rising of sea level [6–8]. Non-optimal temperatures around the world have led to substantial mortality [9]. Climate change can act as an amplifier and can exacerbate stress and a greater vulnerabilities in children, the elderly, pregnant and lactating women and people with disabilities or pre-existing physical or mental health problems, including low-income populations, especially those in lower- and middle-income countries. Additionally, the repercussions of climate change are not evenly distributed, often disproportionately affecting those who bear the least responsibility.

Recognizing the urgency of this issue, numerous agreements have been pursued to address it, including the Paris Agreement, which has as one of its goals to limit the temperature increase to 1.5°C [10, 11]*.* An important outcome of this agreement is the Nationally Determined Contribution (NDC) [12], in which 193 parties have committed to reducing their emissions and adapting to climate impacts.

Shrinking of green spaces, especially in the urban settings, is also now a global phenomenon. Green spaces, parks and amenities have an ameliorative effect on people. Air and water pollution by plastics and chemicals are reducing human well-being and burdening the health systems across the world. A particulate matter (PM) level of PM_2.5_ alone can damage human health (such as through cardio-and-neurovascular dysfunction). Evidence from India, Ghana, Ethiopia and Rwanda also suggests pollution is causing decline in IQ of children [13, 14] The growth of plastics also causes neurotoxic effects and significantly impacts health in general and mental health in particular [15].

## Climate Change and Mental Health

The impact of climate change has been mainly focusing on physical health, such as an increase in vector-borne diseases, non-communicable diseases and respiratory illnesses [16]. However, there is a growing research effort to explore the nexus between climate change and mental health. The Intergovernmental Panel on Climate Change (IPCC) highlights that the rapidly escalating effects of climate change pose a threat to mental health and psychological welfare, resulting in various negative outcomes such as emotional distress, anxiety, depression, grief and even an increased risk of suicide ideation [16].

According to the World Health Organization (WHO), mental health is defined as “a state of mental well-being that enables people to cope with the stresses of life, realise their abilities, learn effectively, work productively, and contribute to their community.” In fact, the WHO motto that “there is no health without mental health” is an important reminder here [4]. Mental health is affected by climate change through direct and indirect pathways. For example, extreme climate events such as heatwaves, flooding and drought increase the risk of post-traumatic stress disorder, depression and anxiety. Climate change leads to adverse mental health outcomes among vulnerable populations and those with pre-existing health problems and also exacerbates health inequalities [4, 17–21]. Planetary health influences mental health, and a better management of climate, biodiversity and pollution would have co-benefits of improving some mental health outcomes.

Mental health, however, has been largely marginalised within environmental and climate change sciences and a greater understanding of the planetary health and mental health nexus is needed to cast light on the complex underlying processes and their societal costs to inform policy responses. Mental health is viewed by the lay audience as a science that focuses on individuals and groups with extreme socio-emotional and cognitive disturbances and disorders. Within various disciplines in biomedical and social sciences it often thought of as a field that focuses on intangible, subjective and sometimes hard-to-quantify processes and problems. It is also debated whether mental health is a social phenomenon. With such perceptions and misgivings about the field, environmental and climate sciences have not given full attention to mental health outside of its documentation as being within ecosystem cultural services. The impacts of large-scale geological changes on human anthropogenic activities, heightened consumption and depletion of natural resources, have not been linked to mental and behavioural health with consistency and thoughtfulness. Similarly, it is only recently that a case for public mental health has been made around conservation of natural resources and benefits of the natural environment to human psychosocial well-being. Due to the slow and limited connection between these fields, practitioners from the two arenas have not had opportunities to enhance conceptual and methodological domains informing integrated interventions. Behavioural health is one speciality within psychological and mental health sciences that has remained underutilised. We would like to recommend greater uptake of behavioural strategies and behavioural health approaches to generate synergies. While fields like behavioural economics have been widely used to address development challenges and policy issues such as taxation, public health behavioural change, electoral behaviour and citizen involvement in public policy. While in environmental and climate sciences, expert-led, regional-level dialogues or consultations on behavioral and mental health strategies have been promoted by funding agencies and scholars from both fields. Though the current interest in connecting the dots between nature and mental health is laudable, leveraging mental health to mitigate climate change impacts and focused identification of adaptation mechanisms for different populations and regions will be critical; and a deeper and more rigorous unification of the two fields needs to happen to achieve this.

## A Way Forward

The environment and health community needs to focus on deepening the fields of environment and health to see synergies in connecting these large-scale changes to short- and long-term mental health while providing pointers to enhanced methodological rigor in inquiries jointly or separately developed within these fields. At the same time, this community needs to build environmental and public policy arguments towards strengthening the application of mental health sciences, framing holistic domains of research and action in planetary health and environmental management.

A meta-analysis of evidence from the mental health field [2] came up with insightful perspectives on the nexus between the two. The evidence on this nexus is emerging now and suggests greater momentum on agreement that the association between post-traumatic stress disorder, depression, anxiety, and suicidality and worsening environmental conditions deserves [2]. Other studies have identified similar findings, including extending a social justice and human rights perspective warning us of large scale inequities not only impacting life on earth broadly but specifically affecting access to food, water, sanitation, health services and development activities [22, 23].

Conventional economic approaches to understand the nexus of planetary health and human health have limitations, and we need quantified studies from diverse contexts and locations. A snapshot study that applied value transfer function of the costs suggests that globally, the additional annual societal costs of mental disorders due to changes in climate-related hazards, air pollution and access to green space are estimated to be almost US$47 billion in 2030 and $537 billion in 2050, relative to a baseline scenario in which these environmental factors remain at 2020 levels [1]. These costs and prevalence of societal costs of mental illness are expected to increase with a country’s income, rise in economic inequality, rise in exposure to natural hazards and air pollution and lack of open access to green space. The mental health consequences of environmental degradation are likely to be further exacerbated in lower-income or environmentally vulnerable settings where there is a combination of environmental pressures, insufficiently resourced health systems and constraints on access to health care [16].

We strongly feel that some of the top priorities to align mental health and planetary crises will become imperative to the climate and conservation community and development practitioners in the long run. In the short run, a few considerations will be necessary to be fulfilled. *First*, there is a need to better understand environmental determinants of health, especially mental health. Increases in temperature, pollution and loss of green space are likely to be dominant drivers of compromised mental health, creating burgeoning economic costs. When this occurs in communities, several types of costs compound for individuals with added vulnerabilities and in countries with significant development challenges. *Second*, policymakers and implementers need to be aware of the combined costs of inaction that might accrue from neglecting mental and planetary health. Connecting the fields of mental and planetary health is important for robust public policy response and long-term environment management. *Third*, improved methodologies are required for studies on the environment and mental health nexus. *Fourth*, global health agencies, local environmental and climate assessments may be under-studying potential trauma and traumatic stress arising from the ongoing and escalating climate crisis [4]. The nosology of trauma in this parlance also needs broadening as environment- and climate-associated psychological, cognitive, emotional and social experiences will be larger than anticipated: more frequent, interconnected, spiralling from one event to the next, transboundary and often larger in magnitude, therefore experienced not only at individual but also at collective levels. The trajectories and models of adaptation and mitigation of climate change need to be informed by mental health and behavioural sciences approaches and recognise the health co-benefits of integrating mental health in adaptation and mitigation. This integration is not yet available in textbooks and playbooks of environmental conservation or adaptation to climate change or in handbooks of psychiatry, mental or behavioural health. This is a field that must be actively curated with local and global actors, working with communities, different disciplines and with a range of expertise from environment, climate and mental health sciences. The earlier we recognise that this dialogue needs to happen to frame interventions and public policy response, the better it will be for humankind. We need multiple types of mental health solutions as people live in diverse geopolitical areas, varying socioeconomic conditions and natural ecosystems. In fact, too many people worldwide live with very little and in politically, environmentally and economically challenging circumstances. *Fifth*, behavioural health researchers need frameworks that cover environmental risks and a more comprehensive understanding of emerging environmental and climate-change associated public health issues. These efforts require improved interdisciplinary collaborations, co-designing interventions and policies with communities, including those in environmental stress, and academic partnerships that integrate policy and practices across the Global North and South. Promoting research priorities of Global South mental health and climate scientists will be an important arena of capacity development. *Sixth*, data and indicators on climate change can benefit from the inclusion of mental health outcomes. *Seventh*, due to deteriorating environmental and climactic conditions, the costs associated with mental ill-health at the global and national levels are likely to worsen; therefore, these inclusive indicators can drive timely action and an integrated response of budgetary planning and program implementation. Finally, meaningful, contextually relevant research that addresses the gaps we have highlighted above will enable policy- and practice-relevant evidence to bridge fields of mental health and climate action, especially for national policymakers.

Future research would benefit from enhanced methodological rigour, stronger study designs and use of multilevel theoretical frameworks around which human well-being, mental health and environmental exposures can be better understood and coordinated interventions can be designed. There is also a need to develop quantitative measures of climate-change-related mental health effects and define and quantify causal pathways, including moderating and mediating factors, between climate change exposures and mental health outcomes [17,24,25]. Additionally, research findings in this area from high-income settings may not be generalisable to differing socioeconomic and environmental contexts [1]. Studies on economic impacts associated with mental illnesses and their societal costs from Australian, European, Japanese and North American contexts may not be relevant for low-resourced African, Latin American and Asian contexts, where environmental problems, economic growth, population density, social determinants of well-being, the manifestation of mental illnesses [26] and health system settings can vary considerably [27].

## Conclusion

The identification of co-benefits of environmental improvement on mental health could motivate global and national decision makers to integrate benefits of environmental considerations in policy responses to address mental ill-health. The consideration of economic impacts in relation to climate change in the 2006 Stern Review [27] and biodiversity in the 2021 Dasgupta Review of 2021 [28] are instructive for this approach. The current global agreements like Kunming Montreal Global Biodiversity Framework [29–32], Convention of Plastics [15], new impetus on Loss and Damage under Convention of Parties of the United Nations Framework on the Convention of Climate Change (UNFCCC) [31] and the recently launched Global Chemical Framework [32] will need to account for how their actions are going to affect the constituents and determinants of mental health. The estimation of costs of mental illnesses due to environmental deterioration would also provide justification for allocation of public and private investment in efforts to address these issues at the national level. Our assessment finds that under the business-as-usual scenario, the costs of range of mental illnesses are substantial and will compromise the productivity of the workforce, burden health-care systems, lower human capital, stall progress towards the UN sustainability targets, deplete environmental resources and threaten our well-being and survival in the long run.

## Data Availability

All data cited here is from publicly accessible sources; no primary data was collected.
